# Bone Loss and Fractures in Post-Menopausal Women Living with HIV: A Narrative Review

**DOI:** 10.3390/pathogens13090811

**Published:** 2024-09-19

**Authors:** Maryam Jamshaid, Amirmohammad Heidari, Ahmed Hassan, Dushyant Mital, Oliver Pearce, Maria Panourgia, Mohamed H. Ahmed

**Affiliations:** 1 Department of Trauma and Orthopaedics, Liverpool University Hospital NHS Trust, Liverpool L69 3BX, UK; maryamjamshaid9819@gmail.com (M.J.); am.heidari5@gmail.com (A.H.); 2 School of Medicine, University of Liverpool, Liverpool L69 3BX, UK; 3 Faculty of Medicine, Alexandria University, Alexandria 21500, Egypt; ahmed.mohamed2133@alexmed.edu.eg; 4 Department of HIV and Blood Borne Virus, Milton Keynes University Hospital NHS Foundation Trust, Eaglestone, Milton Keynes MK6 5LD, UK; dushyant.mital@mkuh.nhs.uk; 5 Department of Trauma and Orthopaedics, Milton Keynes University Hospital NHS Foundation Trust, Milton Keynes MK6 5LD, UK; oliver.pearce@mkuh.nhs.uk; 6 Department of Geriatric Medicine, Milton Keynes University Hospital NHS Foundation Trust, Eaglestone, Milton Keynes MK6 5LD, UK; maria.panourgia@mkuh.nhs.uk; 7 Faculty of Medicine and Health Sciences, University of Buckingham, Buckingham MK18 1EG, UK; 8 Department of Medicine and HIV Metabolic Clinic, Milton Keynes University Hospital NHS Foundation Trust, Eaglestone, Milton Keynes MK6 5LD, UK

**Keywords:** bone loss, fractures, post-menopausal, women, living with HIV

## Abstract

Introduction: Post-menopausal women living with Human Immunodeficiency Virus (WLHIV) face an increased risk of bone fractures due to the relationship between HIV-related factors and menopause. This narrative review aims to summarise the current knowledge about fracture risk among post-menopausal WLHIV in particular looking at hormonal changes, combined antiretroviral therapy (cART), lifestyle factors, and psychosocial implications. We also profiled a summary of the significant, recent studies of post-menopausal WLHIV residing in low-income countries (LIC). Methods: A thorough search of the literature was performed across PubMed, Medline, Scopus, and Google Scholar, focussing on studies published between 2000 and 2024. Inclusion criteria entailed original research, reviews, and meta-analyses addressing bone mineral density (BMD), fracture incidence, and related risk factors in post-menopausal WLHIV. Results: The review identified 223 relevant studies. Post-menopausal WLHIV exhibit significantly lower BMD and higher fracture rates compared to both HIV-negative post-menopausal women and pre-menopausal WLHIV. cART, particularly tenofovir disoproxil fumarate (TDF), contributes to reduced BMD. Menopausal status exacerbates this risk through decreased oestrogen levels, leading to increased bone resorption. Moreover, lifestyle choices such as smoking, alcohol consumption, and low physical activity are more prevalent in PWHIV, which further elevates fracture risk. Different psychosocial factors may make WLWHIV more vulnerable at this stage of their life, such as depression, isolation, stigma, and housing and nutritional issues. Women living in LICs face a variety of challenges in accessing HIV care. There are gaps in research related to the prevalence of osteoporosis and bone loss in post-menopausal WLHIV in LICs. Conclusion: Post-menopausal women living with HIV face a significantly higher risk of bone loss and fractures due to the combined effects of HIV and menopause. Antiretroviral therapy (particularly TDF), lifestyle factors, and psychosocial challenges exacerbate this risk. There is a need for careful selection of cART, hormone replacement therapy (HRT), and emerging treatments such as Abaloparatide. A holistic approach including lifestyle changes and psychosocial support is crucial to reduce fracture risk in WLHIV, especially in low-income countries.

## 1. Introduction

Over the past 25 years, combination antiretroviral therapy (cART) has transformed HIV care from a terminal disease into a chronic condition with normal life expectancy and stability for those who have access to treatment [1]. Before the introduction of combination antiretroviral therapy (cART) in the mid-1990s, HIV was considered a terminal disease. Typically, the virus would progress to AIDS within 10–12 years, and once AIDS was diagnosed, life expectancy dropped to 1–2 years due to opportunistic infections and immune system failure [2]. However, the advent of cART transformed HIV into a manageable chronic condition. For individuals who start cART early, particularly with a CD4 count above 350 cells/mm^3^, life expectancy can be similar to that of the general population. For example, in the UK, a 20-year-old starting cART with a CD4 count between 200–350 cells/mm^3^ has a life expectancy of around 53.4 years, close to the general population’s figures [3].

In addition to the remarkable improvements in life expectancy and mortality, the epidemiology of HIV has evolved to affect females more significantly [4]. In 2023, women accounted for more than half of 39.9 million people living with HIV (PLHIV) globally [5]. In combination with the ageing population increasing globally, the percentage of women over 50 living with HIV is also increasing [6]. Between 2000 and 2024, the trend of HIV affecting women has seen significant shifts [7]. In 2000, women accounted for a lower proportion of global HIV infections, but over time, their share has increased, particularly among young women and adolescent girls aged 15–24 [7]. By 2023, females represented 44% of new global infections, with adolescent girls being four times more likely than boys to contract HIV in this age group. Despite a 63% reduction in new infections among adolescent girls and young women since 2000, progress has slowed in recent years, with 210,000 new cases reported in 2023, compared to a 2025 target of 50,000 [7,8]. 

Biological menopause is a normal part of ageing for women, and this transition has historically been linked to detrimental consequences in women in terms of bone loss [9]. The World Health Organization (WHO) defines biological menopause as the permanent cessation of menses (also known as amenorrhoea) for a period of at least twelve months [10]. While the age of onset of menopause varies with individual factors and geographical location, it typically occurs between the ages of 45–56 for women without HIV [11]. This period of amenorrhoea is caused by non-pathological oestrogen deficiency and a resultant loss of follicular activity in the ovaries, which can increase the risk of bone loss [11]. HIV has been shown to lead to a significantly higher risk of hip and major osteoporotic fractures [12], and thus women living with HIV (WLHIV) experience an intricate, bidirectional relationship between menopause and HIV, which further adds an additional layer of complexity to the natural ageing process. 

WLHIV have also been shown to experience premature menopause. This population is more likely to experience early (before age 45) or premature menopause (before age 40) compared to HIV-negative women. The median age of menopause in HIV-positive women is around 48 years, with early menopause rates ranging from 14.6% to 27.9% and premature menopause rates between 2.3% and 35% [13].

Research on the impact of HIV on ovarian ageing has revealed multiple mechanisms that contribute to early ovarian failure in women living with HIV. Several studies have focused on anti-Müllerian hormone (AMH), a key marker of ovarian reserve, finding that women with HIV generally have lower AMH levels compared to HIV-negative women [14,15]. This suggests a depletion of ovarian reserve, which could indicate accelerated ovarian aging. Lower AMH levels have been linked to chronic inflammation caused by HIV infection. Even with the use of antiretroviral therapy (ART), this inflammation persists [16]. Chronic immune activation, driven by low-level viral replication, microbial translocation from the gut, and ART toxicity, increases the levels of pro-inflammatory cytokines such as IL-1, IL-6, and TNF-α, which damage ovarian follicles and contribute to premature ovarian insufficiency [14,17]. These inflammatory processes and persistent immune activation seem to play a central role in driving early ovarian failure in women with HIV [16].

Moreover, studies have shown that mitochondrial dysfunction, induced by both HIV and long-term ART, accelerates ageing processes within the ovaries [18]. Mitochondrial damage leads to cellular senescence, oxidative stress, and apoptosis, all of which are detrimental to ovarian health [19]. Certain ART medications, such as non-nucleoside reverse transcriptase inhibitors (NNRTIs) and protease inhibitors (PIs), are known to exacerbate this mitochondrial dysfunction, leading to further reproductive ageing [18]. Additionally, as women with HIV transition into menopause, the depletion of oestrogen aggravates immune dysregulation, further increasing the viral reservoirs and immune activation [17].

These hormonal changes are linked to increased inflammation and microbial translocation, which worsen immune responses and lead to further reproductive ageing [19]. Overall, these findings highlight a complex interaction between chronic inflammation, ART-induced toxicity, mitochondrial dysfunction, and hormonal changes, which accelerate reproductive aging and ovarian failure in women with HIV.

In a recent review article, Ahmed et al. concluded that menopause seems to occur at least four to five years earlier in HIV-infected women compared to the general population [20]. Moreover, Chikwati et al. showed that age at menopause in sub-Saharan Africa was lower in WLHIV than in HIV-negative women (48.1 ± 5.1 vs. 50.9 ± 4.7 years, *p* < 0.001) [21]. They also noted that menopause can be associated with metabolic changes such as insulin resistance, dyslipidaemia, and the presence of atherosclerosis. 

A complex clinical process, menopause is a unique experience for each woman, which leads to a wide range of changes in endocrine and other age-related co-morbidities in addition to changes in bone health [22]. HIV has particularly been shown to be associated with evidence of osteoporosis across all menopause stages, unlike those without HIV [23]. In post-menopausal females, the WHO defines osteoporosis as bone mineral density (BMD) measurement taken using dual X-ray absorptiometry (DEXA) at the spine, hip, or forearm that should show a result that is at least 2.5 standard deviations below that of a “young normal” adult (T-score ≤ −2.5), or there should be a history of one or more fragility fractures [24]. While primary osteoporosis can be a normal part of ageing and menopause, secondary osteoporosis is bone loss due to clinical disorders such as endocrine pathology, which often occurs in HIV-infected individuals [25]. Up to 30% of post-menopausal women have secondary osteoporosis [26] and any resultant bone fractures can have devastating consequences on the individual as they can lead to prolonged and costly hospital admissions. Globally, the overall mean one-year mortality rate following a hip fracture is 22.0% [27], which may undermine the improvements in quality of life achieved through cART.

Most research on co-morbidities in PLWHIV has focused on the male population or a combination of both males and females. There has been limited research that specifically addresses bone loss, fractures, and related co-morbidities experienced specifically by WLHIV. Importantly, the concept of effective menopausal care (which constitutes a continuum of symptom management and optimisation of medical health, including cardiovascular, bone, and mental health) is rarely implemented in WLHIV. Dragovic et al., in a recent review on menopause care in the UK, revealed that few HIV specialists are fully trained in menopausal medicine, and general practitioners, or primary care physicians, are cautious when treating menopausal symptoms in WLHIV due to drug-drug interactions with cART and hormone replacement therapies (HRT) [28]. They emphasised that WLHIV experience significant distress due to having to shuffle between specialists to meet their menopausal and HIV needs [28]. Zahn et al. showed there are few interventions that exist to address disparities in menopausal care, even for HIV-negative women [29]. WLHIV thus experience a compounded lack of high-quality, individualised menopause care, which would lead to improved patient experience. It is important to highlight that a diagnosis of menopause with HIV is still dependent on HIV physician judgement due to a lack of consensus. For instance, Scofield et al. demonstrated disparities among HIV guidelines in the diagnosis of menopause and the assessment and treatment of menopausal symptoms [30]. They included five HIV and six general menopause management guidelines published between 2015 and 2023, and five menopausal symptom assessment scales. They found that the HIV guidelines did not offer guidance on how to diagnose menopause or how to differentiate between HIV-related and menopause-related symptoms. Importantly, the scales for menopausal symptom assessment were not validated specifically for WLHIV. The authors recommended the need for the development of a comprehensive guideline that addresses all relevant factors in managing menopause in women with HIV [30].

The aim of this narrative review is to elucidate the effects of bone loss and related co-morbidities of HIV in post-menopausal women, including those living in LICs, and recommend key management areas by drawing on existing research to highlight key findings, mechanisms, and clinical implications. We much hope this review will contribute to establishing an initiative to promote the concept of effective menopausal care among women living with HIV. 

## 2. Methods

**Figure 1:**
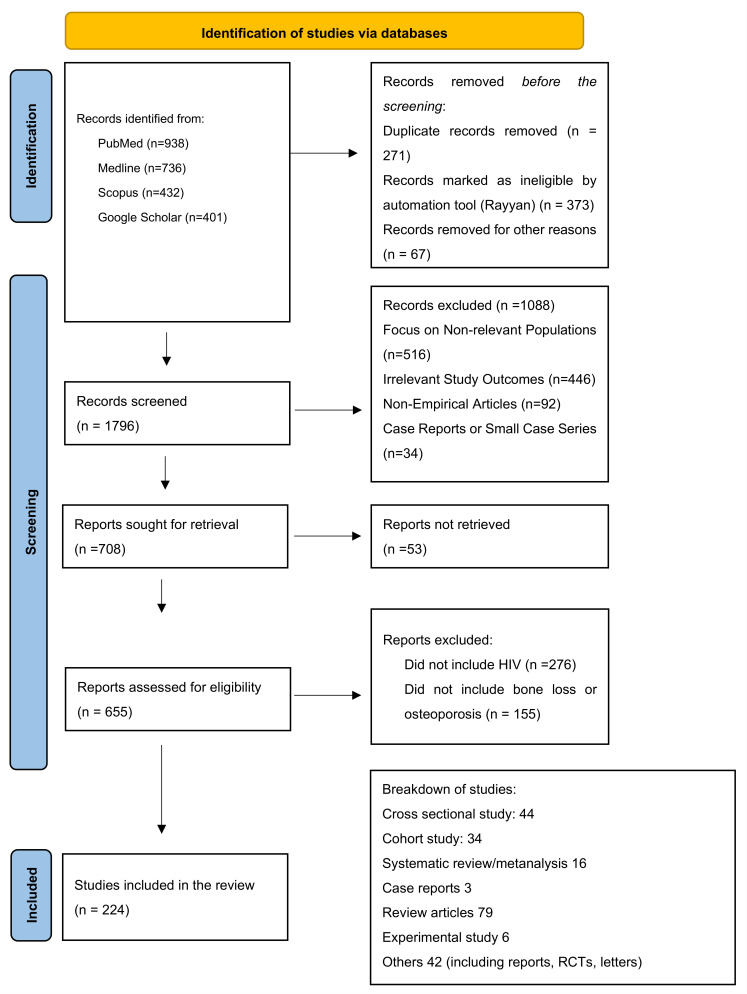
Diagram detailing the identification of studies via databases included in this narrative review.

This narrative review article was conducted by undertaking a comprehensive search across PubMed, Medline, Scopus, and Google Scholar, focussing on studies published between 2000 and 2023. Keywords included “Osteoporosis” OR “Bone density*” OR “Bone loss*”, “Postmenopausal*” OR “Menopause*”, “Risk factor*” OR “Prevent*” OR “Treat*” OR “Calcium” OR “Vitamin D” OR “Frac*”, “HIV” OR “Human immunodeficiency virus” AND “ageing woman” OR “post-menopause” OR “post-menopausal”. Inclusion criteria entailed original research, reviews, and meta-analyses addressing BMD, fracture incidence, and related risk factors in post-menopausal WLHIV. Only articles published in the English language were included. Figure 1 shows how the literature search was conducted and the number of studies included in this review article. In this narrative review, we evaluated a broad range of studies to understand bone loss and fracture risk in post-menopausal women living with HIV. The inclusion and exclusion criteria used in this review are listed below.

### 2.1. Inclusion Criteria

We included studies published between 2000 to 2024.

Study types included: epidemiological studies, clinical trials, systematic reviews and meta-analyses, observational studies, pathophysiological studies, animal studies, and in vitro studies.

Population focus: the primary population was post-menopausal WLHIV, with studies from various geographical regions. The review criteria included papers for comparisons with pre-menopausal WLHIV to highlight the impact of menopause, with HIV-negative post-menopausal women to isolate the effects of HIV and ART on bone health, and with men living with HIV to contextualise gender differences in bone health outcomes.

Relevant studies had the following outcomes of interest:

1. Bone Mineral Density (BMD)
2. Fracture Incidence and Risk
3. Bone Turnover Markers
4. Vitamin D Status
5. Impact of Antiretroviral Therapy (ART)
6. FRAX Score and Fracture Risk Prediction
7. Influence of Co-Morbidities and Lifestyle Factors

### 2.2. Exclusion Criteria

1. Population-specific exclusions:
  ○ Studies not involving women. (Include both pre- and post-menopausal women, but studies focussing only on men are excluded.)
  ○ Studies not including HIV-infected individuals.
2. Outcome-specific exclusions:
  ○ Studies that do not address bone health (such as BMD, fractures, or osteoporosis).
  ○ Research that focusses on unrelated health outcomes (e.g., cardiovascular disease, mental health) without mentioning bone health or fracture risk.
3. Study design exclusions:
  ○ Non-peer-reviewed articles (e.g., conference abstracts, editorials, letters).
  ○ Studies not based on human subjects (exclude animal or laboratory studies).
  ○ Case reports or small case series that do not provide broad or generalisable findings.
4. Geographical or setting exclusions:
  ○ Studies conducted in regions not relevant to the global HIV burden, such as those focused solely on populations where HIV and osteoporosis are not common comorbidities.
5. Language exclusions:
  ○ Studies not published in English.
6. Publication date exclusions:
  ○ Studies published before 2000, to focus on more recent data relevant to current HIV treatments and management of bone health.

To ensure the robustness of our findings, we prioritised higher-quality evidence such as cohort studies, systematic reviews, and meta-analyses. Cross-sectional studies and case reports were included for additional context but recognised as less rigorous. Only studies directly assessing relevant outcomes, such as bone mineral density (BMD) and fracture incidence, were considered, with preference given to those using validated tools such as dual-energy X-ray absorptiometry (DXA) scans. We also prioritised studies that controlled for key confounding factors such as age, body mass index (BMI), menopausal status, and HIV treatment regimen to minimise bias. Geographical and temporal relevance were also key considerations. We placed an emphasis on studies conducted in low-income countries, where the burden of HIV is particularly high, and limited our scope to studies published between 2000 and 2023 to ensure relevance to modern treatment approaches. While we did not formally apply a bias assessment tool, each study was critically appraised for methodological soundness, with particular attention to potential sources of bias, such as selection and reporting biases. This approach ensured that our review provides a comprehensive yet methodologically sound overview of the current evidence.

## 3. Results

### 3.1. Epidemiology

In 2023, women made up 53% of the total 38.4 million PLWHIV [2]. The proportion of women affected by HIV varies by geographical location. Although sub-Saharan Africa (SSA) accounts for 12% of the world population, the region contains 70% of all PLWHIV [31]. In SSA, women and girls account for 63% of all HIV diagnoses [2]. In the UK, women accounted for one-third of all PLWHIV in 2022 [32]. The United Nations Population Fund (UNFPA) has revealed that the global share of people aged 65 and over has nearly doubled from 5.5% in 1974 to 10.3% in 2024 [33]. Due to the increasing ageing population, it is estimated that by 2030, nearly 70% of all PLWHIV will be above the age of 50 in the developed world, thus increasing the proportion of WLHIV who are post-menopausal [34].

This substantial rise in the number of post-menopausal women accessing care in the UK is reflected in the PRIME (Positive Transitions Through the Menopause) study [35]. One of the largest studies globally, the PRIME study approached 1999 women in the UK between June 2015 and April 2018 [17]. It revealed that 10,350 WLHIV, aged between 45 and 56 (and therefore, possibly menopausal), accessed HIV care in 2016, which is a five-fold increase from the number of women in this age range accessing HIV care in 2006 and half the total number of WLHIV who attended HIV care [17]. 

Menopause-associated rapid bone loss leads to a two- to three-fold greater fracture incidence in women [36]. Almost 50% of women above the age of 50 will experience a fracture [37]. A recent systematic review has demonstrated a varying prevalence of osteoporosis in post-menopausal women, ranging from 7% to 84% in WLHIV and 0.7% to 23% in HIV-negative women [38]. This wide range reflects differences in study populations, ART exposure, lifestyle factors, and regional disparities. The highest prevalence of osteoporosis (84%) in WLHIV is approximately 3.65 times higher than the maximum prevalence observed in HIV-negative women (23%). This significant difference likely stems from HIV-related chronic inflammation, ART exposure, and other contributing factors that exacerbate bone loss in WLHIV, particularly during menopause. The review also highlighted that post-menopausal WLHIV report a fracture prevalence 1.5 times greater than their non-HIV infected counterparts [38]. Moreover, results from the Women’s Interagency HIV Study showed that HIV infection and menopausal stage were independent predictors of lower BMD and had an additive effect on the lumbar spine and total hip BMD [39]. Similarly, Leite-Silva et al. showed that WLHIV in Brazil are more likely to have osteopenia/osteoporosis and a high FRAX index score [40].

To reconcile the variations between these studies, the data highlight that regional factors, ART regimen, and lifestyle differences contribute to the variability in osteoporosis prevalence and fracture risk in WLHIV. The findings from the Women’s Interagency HIV Study suggest that HIV’s impact on bone mineral density may vary across populations, and further research is needed to explore these discrepancies. More research is needed to fully understand the additive effects of HIV and menopause on bone health across diverse WLHIV populations [41]. Table 1 summarises several studies examining the risk of fractures in post-menopausal women living with HIV (WLHIV). The findings show that WLHIV have a higher risk of fractures, particularly in the lumbar spine, femoral neck, and hips, compared to non-HIV-infected women. One study found that WLHIV had a 6.3% greater chance of vertebral fractures and lower bone mineral density (BMD) at the femoral neck and hip, with 22 out of 104 participants diagnosed with osteoporosis but not receiving treatment. Another study revealed that older HIV-positive women were 2.11 times more likely to suffer hip fractures compared to non-HIV women. Additionally, a Brazilian study indicated that WLHIV are more prone to osteoporosis and have a higher FRAX score, which estimates the risk of major osteoporotic fractures. Overall, Table 1 highlights the increased fracture risk in WLHIV, emphasising the importance of monitoring bone health in this population to prevent fractures.

It is important to note that PLWHIV experience greater postoperative complications following surgical treatment for the above examples of fractures. Anand et al. showed that due to decreasing CD4+ counts, PLWHIV experience poorer immunity and are more likely to contract opportunistic infections in the postoperative period [46]. Furthermore, in a retrospective study of 37 patients, Akkaya et al. demonstrated that the in-hospital complication rate for PLWHIV following total knee arthroplasty (TKA) or total hip arthroplasty (THA) was 10.8% (complications included delirium, acute renal failure, deep vein thrombosis, and trochanteric fracture) [47]. In contrast, a recent systematic review revealed a systemic medical complication rate of 5.1% following THA and 6.9% following TKA in non-HIV-infected patients [48].

### 3.2. Pathophysiology

In this section, we will discuss normal bone loss and the pathophysiology of HIV and its direct and indirect effects on systems that lead to bone loss. The pathophysiology of HIV directly impacts the immune system and indirectly impacts reproductive hormones, metabolic disease, and thyroid hormones. We will then discuss how patients with HIV are often concurrently found to have nutritional deficiencies and co-infection with Hepatitis C, which also contributes to bone loss, especially in post-menopausal WLHIV. 

#### 3.2.1. Normal Bone Loss Due to the Ageing Process

**Figure 2:**
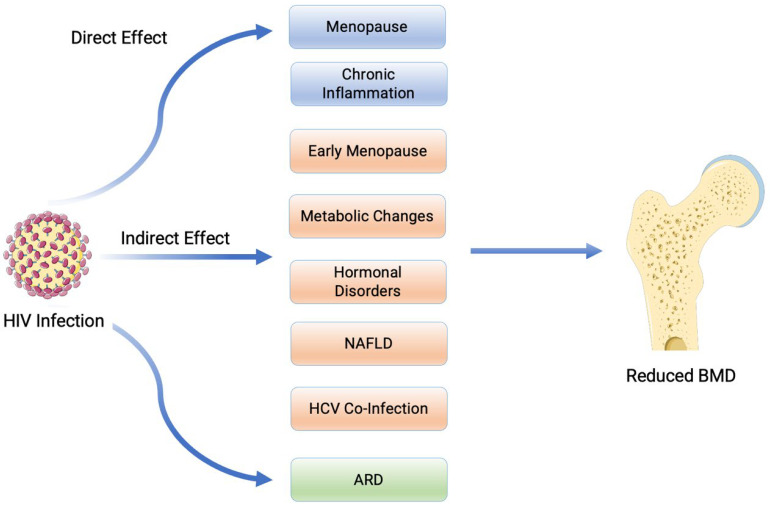
The direct and indirect impact of HIV on physiological systems leads to bone loss. Abbreviations used in the figure: BMD: Bone Mineral Density, NAFLD: Non-Alcoholic Fatty Liver Disease, HCV: Hepatitis C Virus, ARD: Antiretroviral Drugs.

Following peak bone mineral density in early adulthood (25–35 years of age), the skeleton undergoes constant bone remodelling in both men and women [49]. Skeletal remodelling is homeostatic, with osteoclasts (causing bone resorption) and osteoblasts (synthesising new bone cells) in harmony with each other [50]. Giant multinucleated cells of hematopoietic (myeloid) origin, osteoclasts resorb bone under the coordinated function of two key cytokines, the receptor activator of NF-κB (RANKL) and osteoprotegerin (OPG) [51]. RANKL is a key cytokine that drives osteoclast differentiation and subsequent activity. OPG acts as a decoy receptor for RANKL; thereby, it moderates bone resorption [52]. Therefore, the rate of bone resorption in the body is determined by the ratio of RANKL and OPG [52]. Normal ageing leads to an increase in OPG by B-cells to target excessive RANKL activity, which, if left unchecked, may lead to high rates of bone resorption [53]. Despite the increase in OPG, ageing, overall, leads to a gradual loss of BMD as the rate of new bone formation drops below the rate of resorption [49]. Over four to eight years, post-menopausal women experience a 20–30% loss of trabecular bone and 5–10% loss of compact bone [51]. This is crucial, as a remarkable number of WLHIV experience early menopause. Importantly, direct factors such as the HIV virus and menopause will have a substantial impact on increasing bone loss in the majority of WLHIV. In terms of indirect factors (diabetes, thyroid disorders, fatty liver, and hepatitis), even though they are commonly associated with HIV, not all WLHIV will encounter them. In addition, HIV medication per se is also known to be associated with bone loss. In the following sections, we attempt to provide a summary of all these factors. A brief summary of the direct and indirect effects of HIV on reduced BMD is demonstrated in Figure 2 below. 

**Direct impact of HIV on bone loss.** HIV per se as a cause of ongoing chronic inflammation: PLWHIV have historically been known to demonstrate lower BMD [49]. A pooled odds ratio of 3.7 has been demonstrated by a recent meta-analysis that compared BMD in HIV-infected versus non-HIV-infected persons, thereby highlighting the accelerated rate of bone loss in PLWHIV [10]. Another study showed that people living with HIV (PLWHIV) have a higher risk of reduced BMD and fractures due to HIV-induced chronic inflammation and the effects of antiretroviral therapy (ART) [54]. Similarly, a review showed that PLWHIV experience accelerated bone loss, with additional contributions from lifestyle and nutritional factors. These studies collectively highlight the need for focused bone health monitoring in PLWHIV [55].

The exact causes and mechanisms of bone loss directly due to the virological nature of HIV remain unknown. However, risk factors contributing to accelerated bone loss have been demonstrated in HIV-infected communities, including social factors such as smoking and excessive alcohol, as well as HIV-related effects such as low BMI, hypogonadism, and kidney disease [56]. PLWH is a particularly vulnerable cohort prone to problematic health behaviours and are more likely to drink alcohol and use drugs [57]. The immune-skeletal interface (ISI) is a complex system of shared cells and cytokines through which the immune system directly impacts the skeletal system [58]. In the context of HIV infection, the chronic inflammation diminishes the compensatory increase in OPG that normally occurs during ageing, thereby leading to increased RANKL production. This may lead to an exacerbated loss of bone [58]. HIV-infected T-cells can modulate the function of macrophages and lymphocytes and, most importantly, enhance the recruitment of osteoclasts, leading to an increase in RANKL and low expression of OPG [59]. Recent research into the molecular pathways has revealed that HIV-infected T-cells and B-cells, specifically those with prolonged viral exposure, cause persistent upregulation of RANKL, particularly through the production of soluble RANKL by activated T-cells. This soluble form of RANKL plays a more significant role than previously understood in directly stimulating osteoclast precursor cells, contributing to sustained osteoclastogenesis in PLWHIV [60,61]. A five-fold increase in RANKL production from B-cells was observed in HIV patients compared to non-HIV controls, leading to amplified osteoclast differentiation and bone resorption. Furthermore, the reduction in OPG in these individuals, exacerbated by chronic HIV-driven immune dysregulation, significantly contributes to this imbalance, favouring excessive bone breakdown [62].

Unfortunately, additional cellular processes continue to erode the bone; for example, the osteoclast in HIV activates RANKL, tartrate-resistant acidic phosphatase (TRAP), and cathepsin K, and this leads to more osteoclast activity, thus decreasing bone density [63]. Molecular studies have shown that TRAP and cathepsin K expression is significantly upregulated in PLWHIV, further intensifying osteoclast-mediated bone matrix degradation. The upregulation of these key enzymes results in more aggressive bone resorption, contributing to a heightened loss of bone mineral density and overall skeletal fragility [64,65]. Besides decreasing the osteoblasts or osteocalcin, HIV modulates the bone marrow mesenchymal stem cells, and this leads to a release of interleukin-6 (IL-6), interleukin 8 (IL-8), alkaline phosphatase (ALP), runt-related transcription factor 2 (RUNX-2), and bone morphogenic proteins (BMP-2, BMP-7) [66]. Chronic inflammation plays a central role in HIV-induced bone loss. Elevated levels of pro-inflammatory cytokines, especially IL-6 and TNF-α, have been identified as major contributors to increased bone resorption. IL-6, which is upregulated in PLWHIV, directly correlates with reduced BMD and stimulates the differentiation and activation of osteoclasts by increasing RANKL expression. IL-6 also disrupts osteoblast activity, inhibiting bone formation, thus further exacerbating bone loss [60]. 

All of these factors work toward decreasing bone formation and increasing osteoclastic activities (bone resorption) [67] Studies on HIV-transgenic rats, an animal model of HIV infection that lacks many of the confounding influences on bone that are associated with human HIV patients revealed a significant 75% decline in expression of bone marrow OPG due to defective B cell OPG production [67] This loss of OPG, coupled with a dramatic five-fold increase in receptor RANKL production by B-cells, accounted for a significant increase in osteoclast differentiation [67]. Overall, In vivo markers of bone resorption increased by 43%, while bone formation markers remained unchanged. This increase in the rate of bone resorption led to a significant loss of BMD, which was demonstrated using DXA, with a percent change between HIV transgenic rats and control groups of −36% in femurs, −29% in tibias and −21% in the lumbar spine. Moreover, cortical volume of bones was also significantly reduced by −18.7% [67]. In the context of women and menopause, both B-cells and T-cells have been shown to be key sources of enhanced RANKL production in post-menopausal women relative to premenopausal women and post-menopausal women receiving oestrogen replacement [68].

Overall, as described above, the highlighted cascade of events illustrates how HIV worsens bone health by enhancing bone resorption. HIV stimulates mesenchymal stem cells to release cytokines (IL-6, IL-8), ALP, and BMPs, promoting osteoclast activity while reducing bone formation. In HIV-transgenic rats, a 75% reduction in OPG and a five-fold increase in RANKL significantly increased osteoclast differentiation, leading to a marked decrease in BMD (36% in femurs, 29% in tibias). This is critical for PLWHIV, especially post-menopausal women, as it accelerates bone loss and increases fracture risk. (Figure 2)

**Gonadal hormones and effects on age at menopause.** Among HIV-infected women, early menopause is frequent [20]. Serum plasma levels of anti-Mullerian hormone (AMH), an indicator of ovarian reserve and FSH, are highly predictive of the age onset of menopause [69]. In WLHIV, AMH has been documented to be significantly lower, directly associated with the early onset of menopause [69,70]. Another study found that AMH is a strong predictor of the timing of the final menstrual period in HIV-infected women [71]. However, other factors can additionally cause early menopause, such as low BMI, ethnicity, smoking, low CD4+ cell count, hepatitis C virus coinfection, and a history of Acquired Immunodeficiency Syndrome (AIDS) [20,72] The mechanism of this correlation is multivariant, as one of the most prevalent predictors of early onset of menopause is the use of tobacco inhalation inhalation [51]. Smoking is common among WLHIV and is often accompanied with the use of recreational drugs [73]. The use of cocaine and heroin five years before the onset of menopause is associated with early-onset menopause [74]. 

A recent survey of 102 WLHIV showed that the age of menopause in WLHIV ranged between 39 and 49 years (median age: 46), while it was between 44.5 and 48 years (median age: 47) in uninfected women [74].

Ethnicity, in particular, has also been shown to play a role in early menopause in WLHIV. A retrospective Swiss study demonstrated that Black women were found to be four times more likely to experience early menopause than women of other ethnicities (aOR = 4.2, 95% CI: 2.5–7.2, *p* < 0.001) [72]. Interestingly, traditional risk factors such as smoking, drug use, and body weight did not significantly impact the age of menopause, indicating that ethnicity plays a dominant role [72]. HIV-related factors such as low CD4 counts, viral suppression, or hepatitis coinfection also showed no effect. Being underweight was initially associated with a later menopause but was excluded due to confounding with Black ethnicity. Despite Black women being more likely to be overweight (70% vs. 32%) and less likely to smoke (10% vs. 57%) or use drugs (1% vs. 20%), they still experienced earlier menopause [72]. This highlights that biological factors and possibly socioeconomic disparities are stronger determinants of early menopause in this population than lifestyle factors [72].

Additionally, a study in Burkina Faso with 93 patients revealed that early menopause occurred in 5.5% of WLHIV but only occurred in 1.4% of a non-infected African population [75].

**Indirect impact of HIV on metabolic disorders and bone**. Diabetes, dyslipidaemia, and obesity. The menopause transition leads to increased risk of cardiovascular disease (CVD) due to factors such as the loss of the protective effects of oestrogen. Lipid profile changes that lead to atherogenesis, such as increased low-density lipoprotein (LDL), total cholesterol, and apoprotein B, have been noted in peri-menopausal and post-menopausal women [76]. These factors have been independently associated with abdominal fat, age, and menopause [76]. Reduced HDL levels and increased insulin resistance due to changes in androgen levels have been exhibited during menopause [77]. This leads to an increased risk of developing type II diabetes, non-alcoholic fatty liver disease (NAFLD), metabolic associated fatty liver disease (MFFLD), and metabolic syndrome [78]. 

For individuals with HIV who are not on ART, there is a decrease in total cholesterol (TC), LDL cholesterol (LDL-C), and HDL cholesterol (HDL-C) compared to pre-seroconversion levels, while triglycerides (TG) show no significant change compared to individuals without HIV [79,80]. When people living with HIV begin ART, TC and LDL-C levels tend to increase compared to pre-ART levels. HDL-C generally shows no change, and the effect on TG levels varies depending on the type of ART being used [80,81]. In cases of advanced HIV or AIDS, lipid levels behave differently. There is a decrease in TC, LDL-C, and HDL-C compared to individuals without HIV, while TG levels tend to increase, particularly when compared to both individuals without HIV and those with HIV but without AIDS [79,80]. Lipodystrophy, particularly in patients undergoing ART, contributes significantly to these metabolic disturbances. The redistribution of fat (e.g., loss of subcutaneous fat and increased visceral adiposity) promotes a chronic inflammatory state, marked by increased TNF-α and IL-6, which not only worsens insulin resistance but also disrupts bone health by increasing bone resorption and reducing bone formation. This process elevates the risk of osteopenia and osteoporosis in PLWHIV [82,83,84].

PLHIV are at a higher risk of CVD, hypertension, and diabetes due to multiple factors, such as specific combination antiretroviral therapy (cART) medications, inflammatory effects of HIV, and traditional risk factors such as dyslipidaemia [85,86]. Additionally, risk factors such as smoking and poor fitness levels, which increase the risk of CVD and associated metabolic disorders, are more common in PLWHIV [87,88]. PLWHIV are known to be in a hypermetabolic state with an elevated resting energy expenditure mediated by IL-1, TNF, and interferon-α. 

Insulin resistance, commonly associated with HIV-related lipodystrophy, further exacerbates bone loss. The reduced insulin sensitivity impairs the anabolic effects of insulin on bones, leading to increased osteoclast activity and a weakening of bone integrity. This is particularly concerning in PLWHIV, who often experience a combination of chronic inflammation, visceral fat accumulation, and dysregulated glucose metabolism, contributing to accelerated bone degradation [89,90,91].

Normal growth hormone (GH) levels are essential for maintaining protein balance and muscle mass. Patients with advanced HIV- and AIDS-related wasting type syndromes routinely have elevated GH and low insulin-like growth factor (IGF)-1 levels, suggestive of GH resistance. Testosterone levels also correlate closely with lean muscle mass as WLHIV tend to display lower testosterone levels, leading to a loss of muscle mass and a trajectory towards adipose tissue gain [28]. 

The chronic inflammation seen in PLWHIV not only leads to metabolic alterations but also directly affects bone health. ART, particularly protease inhibitors, exacerbates these issues by inducing lipodystrophy and increasing the production of inflammatory cytokines such as IL-6 and TNF-α. This inflammation interferes with normal insulin receptor function, worsening insulin resistance and contributing to reduced bone mineral density (BMD) [89].

CVD, insulin resistance, diabetes, hypertension, and NAFLD have all been individually linked to low bone turnover and osteoporosis [92,93,94,95,96]. Perimenopausal women have been shown to experience an increase in fat mass and waist circumference, as well as a decrease in skeletal muscle mass [97]. A study that analysed perimenopausal women over a period of six years demonstrated a cumulative increase of 3.4 kg of fat, a 5.7 cm increase in waist circumference, and a 0.23 kg reduction in skeletal mass [97]. These changes were associated with an increase in follicle-stimulating hormone (FSH) during the peri-menopause transition [97]. These modifications experienced by women during the menopause period have been linked to dyslipidaemia, an increased risk of CVD, and insulin resistance [98]. Similar changes are seen in PLWHIV with lipodystrophy, which affects up to 50% of patients. This syndrome is associated with a redistribution of fat, particularly an increase in visceral fat and a reduction in subcutaneous fat, alongside insulin resistance, which not only heightens cardiovascular risk but also contributes to bone deterioration through mechanisms such as increased osteoclast activation [83,89].

A similar experience of fat re-distribution is experienced by PLWHIV, known as lipodystrophy syndrome, where there is an increase in truncal adiposity and a decrease in muscle mass [99]. Up to 50% of all PLWHIV experience such changes [99]. WLHIV have been shown to be more likely to undergo an increase in truncal visceral adipose tissue as compared to non-infected women (age range: 33–45 years) [100]. Moreover, high levels of total visceral adipose tissue have been linked to higher triglyceride levels and lower high-density lipoprotein (HDL) levels in WLHIV [101]. 

Importantly, physical activity has been shown to improve bone mass in PLHIV and is encouraged in clinical settings. A recent randomised control trial (RCT) demonstrated that combined resistance and aerobic training led to significant increases in BMD in the lumbar spine, femoral neck, and radius (all *p* < 0.05). Therefore, post-menopausal WLHIV should be encouraged to engage in exercise to mitigate bone loss [102].

Notably, the gut microbiome imbalance in people living with HIV (PLWHIV) has been shown to contribute to insulin resistance, dyslipidaemia, and chronic low-grade inflammation, which are central components of metabolic syndrome. This microbial dysbiosis alters the production of short-chain fatty acids (SCFAs) and bile acid metabolism, leading to increased lipogenesis (fat formation) and impaired glucose regulation. The combination of microbial translocation from the gut and systemic inflammation also exacerbates insulin resistance and promotes the development of atherosclerosis, increasing the risk of cardiovascular diseases in PLWHIV [103].

**Non-alcoholic fatty liver disease (NAFLD).** A cross-sectional study of 89 PLWHIV on TDF-based cART for over three years found that patients with NAFLD had worse bone mineral density (BMD) than those without NAFLD. The rates of osteopenia (42.86% vs. 25.93%) and osteoporosis (17.14% vs. 3.70%) were significantly higher in PLWHIV with NAFLD. After adjusting for confounders, the odds ratio for poorer BMD in NAFLD patients was 4.49 (95% CI 1.42, 14.15), suggesting a strong association between NAFLD and decreased BMD in PLWHIV [104].

Only a limited number of studies have exclusively analysed the risk of metabolic diseases in WLHIV. After accounting for age, ethnicity, and weight, WLHIV were found to have higher levels of triglyceride, C-reactive protein (CRP), insulin, IL-6, and adiponectin compared to non-HIV infected women [105]. Following the collection of both male and female data, one study exclusively analysed the female population for the risk of presentation with a myocardial infarction (MI). It revealed that female subjects exhibited acute MI rates that were significantly higher (*p* < 0.0001) in WLHIV compared to non-HIV-infected women [86]. Furthermore, the study also demonstrated that the rate of MI increased considerably with age [86], thus suggesting that menopausal WLHIV are at a higher risk of presenting with an MI. 

As PLHIV are already at a higher risk of developing metabolic diseases, post-menopausal WLHIV are at an additionally higher risk of developing metabolic disease due to the superimposed increased risk of developing co-morbidities because of age and hormonal changes, which may subsequently lead to bone loss [78]. 

**Nutritional deficiencies (vitamin D and calcium).** Vitamin D is crucial in calcium absorption in the gut, which maintains normal bone homeostasis [106]. Deficiency in vitamin D is defined as serum 25-OH vitamin *D* < 50 nmol/L (20 ng/mL) [107]. Between 24 and 81% of PLHIV are deficient in vitamin D [108]. The variance in reporting is due to differences in cut-offs used to define deficiency in vitamin D, different geographical locations, varying proportions of patients with darker skin tones, and differences in cART use [109]. A recent meta-analysis of 15 studies revealed that PLWHIV are more likely to be deficient in vitamin D as compared to the general population [110]. Increasing age, male sex, lower BMI, and treatment with cART were shown to be risk factors for vitamin D deficiency [110]. Patients with darker skin tones and of African descent have been shown to have lower vitamin D levels [111]. The literature suggests that despite abundant sunlight, vitamin D deficiency is prevalent in tropical regions due to several factors. Urban residents often spend more time indoors, reducing sun exposure, and air pollution blocks UVB rays needed for vitamin D synthesis. Cultural practices, such as wearing body-covering clothing, limit skin exposure, particularly in Muslim-majority areas. Additionally, people with darker skin tones have more melanin, which lowers vitamin D production [112,113,114].

Moreover, traditional risk factors, such as malabsorption due to HIV-related changes in the gastrointestinal tract, obesity, decreased mobility, and sun exposure, have been linked to lower vitamin D [110]. cART regimes, including efavirenz and protease inhibitors, as well as chronic inflammation due to HIV infection, have been linked to a decrease in vitamin D [115]. Hip and spine BMD reductions were less in patients on cART who were treated with calcium (1000 mg/day) and high-dose vitamin D (4000 IU) at the start of an efavirenz-/emtricitabine-/TDF-based regimen compared to the placebo group [109]. This finding highlights the necessity of stopping bone demineralisation and treating osteoporosis in PLWHIV. Notably, despite efavirenz’s usage, the positive impact of vitamin D supplementation was seen [109].

Recent studies have shown that vitamin D supplementation, particularly in high doses, can have a positive impact on BMD in HIV patients. High-dose vitamin D supplementation (e.g., 20,000 IU weekly) combined with calcium has been shown to reduce musculoskeletal complications and improve overall bone health in patients on antiretroviral therapy (ART), particularly those taking tenofovir or efavirenz, which are known to negatively affect bone metabolism [116]. Another study confirms that vitamin D supplementation can improve BMD, especially in those with significant vitamin D deficiency, although outcomes depend on the dosing regimen and duration of supplementation [117]. In contrast, low-dose supplementation (e.g., 800 IU/day) has been less effective in significantly improving BMD, even though it raises serum vitamin D levels [118]. Furthermore, longer intervention periods (beyond six months) are generally required to see meaningful changes in BMD [117]. In some cases, combining vitamin D with other bone-protective therapies, such as bisphosphonates, might be necessary to achieve optimal results [116,118].

HIV and undernutrition have a reciprocal link. They have been widely documented in PLWHIV [119]. Both play a role in the increasing deterioration of the immune system [119]. HIV diminishes adequate nutritional status, and inadequate nutrition weakens PLWHIV immune systems [119]. Poor nutrition leads to a decrease in minerals such as magnesium, phosphorus, and calcium, which are the primary building blocks of teeth. According to Wadhwa et al., WLHIV are likely to have fewer teeth *(p* = 0.001), thus feeding into the cycle of poor nutritional intake [120]. 

**Thyroid disease in HIV patients**. HIV and thyroid disease leading to bone loss HIV infection can impact the endocrine system, including thyroid function, through direct and indirect mechanisms [121]. The virus itself, cART, and the chronic inflammatory state induced by HIV all contribute to potential thyroid dysfunction [85]. Common thyroid abnormalities in HIV-infected individuals include subclinical hypothyroidism, overt hypothyroidism, euthyroid sick syndrome, overt hyperthyroidism, and Grave’s disease [122]. cART, while lifesaving, has been implicated in both the development and exacerbation of thyroid disorders due to its effects on immune function [121].

In HIV patients, both subclinical and overt hypothyroidism are common, with an increased prevalence linked to chronic inflammation and antiretroviral therapy. Hypothyroidism slows down bone remodelling, causing older bone tissue to accumulate, which can lead to reduced bone density and an increased risk of fractures. In subclinical hypothyroidism, particularly in women, reduced BMD in the hip and spine has been reported, increasing the risk of osteoporosis [123,124].

HIV patients, particularly those with hyperthyroidism, are at risk of accelerated bone resorption, where bone breakdown occurs faster than bone formation. This process, exacerbated by the increased metabolic demands of hyperthyroidism, leads to significant bone loss and a heightened risk of osteoporosis and fractures. Subclinical hyperthyroidism, with suppressed TSH levels, also predisposes patients to fractures, especially in the femoral neck [123,124]. 

Grave’s disease: This autoimmune condition, seen in PLWHIV due to immune system dysregulation, accelerates bone turnover, leading to osteopenia and osteoporosis. Excessive thyroid hormone levels increase osteoclast activity (bone resorption), contributing to substantial bone loss and an increased risk of fragility fractures [124]. 

Thyroxine-binding globulin (TBG) levels have been shown to be elevated in PLWHIV and show inverse correlation with CD4+ counts [125]. Patients with a longer HIV diagnosis have most frequently exhibited hypothyroidism [126]. Low levels of tetraiodothyronine (T_4_), triiodothyronine (T_3_), and reverse triiodothyronine (rT3) have been shown in advanced HIV disease [127]. However, the elevated reverse T_3_ levels characteristic of euthyroid sick syndrome have not been linked to advanced HIV disease [127]. This relationship becomes even more complex in post-menopausal women, who already experience higher incidences of hyper- and hypothyroid disease due to a decline in oestrogen levels, which affects the levels of TBG and, in turn, free thyroid hormone levels [128]. 

Both hyper- and hypothyroidism lead to a net loss of bone. Overt hypothyroidism causes decreased osteoclastic and osteoblastic activity, leading to a prolonged bone remodelling cycle [129]. In contrast, hyperthyroidism leads to an accelerated bone remodelling cycle, which prevents the adequate formation of new bone cells [129]. T_3_ has been directly linked to the action of osteoblasts on chondrocytes. However, its effect on osteoclasts remains unclear [130]. Thyroid disease in PLWHIV has been linked to the chronic stress response as a result of prolonged viral infection that intricately impacts the hosts’ immune systems [127]. However, the associated co-morbidities of HIV infection and its treatments have shown clearer mechanisms of thyroid dysfunction. Opportunistic pathogens and neoplasms can invade endocrine organs and cause hormone dysfunction. For example, Kaposi’s sarcoma (KS) and lymphoma can infiltrate the thyroid gland, leading to destruction (and subsequent hypothyroidism) or enlargement [131]. In particular, *Pneumocystis jiroveci* and *Cryptococcus neoformans* can invade the thyroid gland and cause inflammatory thyroiditis, resulting in either hyper- or hypothyroidism [132]. Moreover, some cART regimes can complicate thyroid dysfunction further. 

Conditions such as subclinical hypothyroidism and overt hyperthyroidism have been reported more frequently in patients on long-term ART. Specifically, older regimens, such as those including efavirenz and nevirapine, are more likely to be associated with these thyroid abnormalities. The chronic inflammation caused by HIV, combined with ART, disrupts the endocrine system, leading to thyroid issues, which in turn can exacerbate bone loss due to impaired calcium metabolism [133]. While access to newer ART drugs, such as dolutegravir, is increasing, the use of older and more accessible regimens in low-income countries continues to be a factor in these thyroid-related complications. Studies suggest that thyroid dysfunction contributes to the metabolic and bone health issues commonly observed in PLWHIV in these regions, making it essential to monitor thyroid function as part of comprehensive HIV care [134].

For example, rifampin, phenytoin, ketoconazole, and ritonavir can induce hepatic microsomal enzymes and cause alterations in the clearance of thyroid hormones [135]. A recognised treatment of KS, interferon-Alpha, has also been shown to cause autoimmune thyroiditis [135].

The intricate links between menopause and thyroid dysfunction leading to bone loss in WLHIV have not been explored in clinical studies. However, a meta-analysis study exploring the outcomes of patients with differentiated thyroid carcinoma demonstrated that post-menopausal women in the general population experience significantly greater loss of BMD compared to pre-menopausal women and men [136]. This may demonstrate that the additional immunological burden of HIV in post-menopausal women and the increased risk of thyroid dysfunction due to multi-fold pathology may lead to increased loss of BMD. 

**Hepatitis C virus (HCV) in HIV patients.** Approximately 6.2% of all PLWHIV worldwide are co-infected with HCV [137]. HCV coinfection, for example, is linked to a 1.6-fold higher risk of fractures due to its detrimental impact on bone health and accelerated liver disease progression [138,139]. This has been corroborated by two meta-analyses that have consistently shown that HCV co-infection increases the risk of osteoporosis in PLWHIV. Dong et al. evidenced that the pooled incidence risk ratio of overall fracture risk in HIV/HCV con-infected individuals was 1.77 (95% CI 1.44–2.18) [139]. Similarly, O’Neill et al. showed that low BMD was increasingly prevalent among co-infected patients compared to HIV mono-infected controls (pooled OR 1.98, 95% CI 1.18, 3.31) [140]. A study in Taiwan analysed a total of 636 menopausal women aged 45–80 years using multivariable regression analysis to explore the link between menopause, BMD, and HCV infection [141]. It revealed that BMD was significantly lower in the HCV-seropositive participants in different anatomic locations than in seronegative individuals (lumbar spine: −1.5 vs. −1.1; total hip: −0.9 vs. −0.6; femoral neck: −1.2 vs. −1.0; *p* < 0.05). HCV-seropositive subjects had higher rates of major osteoporotic fractures (11.3% ± 7.6%vs 9.0 ± 6.8%; *p* < 0.001) [141]. These findings may be explained by González-Reimers et al.’s study, who found that sclerostin levels are higher in chronic HCV-infected post-menopausal patients with lower BMD [142]. Secreted by osteocytes, sclerostin is negatively correlated with bone turnover and osteoporosis in post-menopausal women [143]. Carlo et al. recently concluded disease progression in HIV/HCV co-infection is impacted by vitamin D deficiency and is a risk factor for osteoporosis and resultant bone fractures [144]. Moreover, the interaction between the immune system and the two viruses may cause the upregulation of RANKL and OPG pathways, resulting in bone loss [104,105]. 

**Bone loss due to use of cART.** PLWHIV who are being treated with cART have an increased prevalence of reduced bone mineral density and osteoporosis than untreated HIV patients [106,107,108]. Along the same vein, individuals with HIV and HCV co-infection have been shown to have low Vitamin D levels, thus leading to an increased risk of bone loss [145]. Increased RANKL-mediated bone resorption may lead to a sudden decrease in BMD and lead to the resultant increased prevalence of osteoporosis in PLHIV [146,147,148]. Long-term TDF therapy in HIV and HBV infections is associated with increased loss of BMD [149]. Switching to a non-TDF-based regimen can improve BMD [149]. Decreases in BMD have also been corroborated in HIV-negative patients who take TDF for HIV prevention prophylaxis [111]. The rate of bone loss is the highest during the first one to two years after cART initiation and approaches the degree observed in patients using other medications, such as aromatase inhibitors or glucocorticoids [148].

Efavirenz can lead to decreased serum vitamin D levels via its effects on vitamin D metabolism and binding proteins, leading to bone loss [148]. However, with long-term cART and viral suppression, BMD may increase and stabilise over time [148]. TDF has been shown to cause higher loss in BMD as compared to other types of ART [150]. Cidofovir, an antiviral used to treat cytomegalovirus (CMV) infection with viremia, can cause proximal renal tubulopathy with resulting hypocalcaemia, hypophosphatemia and greater decrease in [151]. Foscarnet, another antiviral also used to treat CMV, can cause hypocalcaemia, hypomagnesaemia, and hypokalaemia through its effects on renal tubular absorption [152]. The combination of foscarnet and pentamidine, a medication used to prevent and treat *P. jiroveci* pneumonia, can cause severe hypocalcaemia [153]. 

AIDS-related lymphoma, certain Mycobacterial strains, or *C. neoformans* infections can cause hypercalcemia due to increased extrarenal activation of vitamin D by activated macrophages or tumour cells [154]. Occasionally, hypercalcemia may occur after initiating cART as part of an immune reconstitution syndrome [154].

A systematic review and meta-analysis highlighted significant bone mineral density (BMD) loss in people living with HIV (PLHIV) following the initiation of antiretroviral therapy (ART). Within the first year of ART, average BMD reductions were 2.68% at the lumbar spine (LS) and 2.66% at the total hip (TH). Notably, the analysis showed that tenofovir disoproxil fumarate (TDF) was associated with more substantial BMD loss compared to tenofovir alafenamide (TAF). Over 48 and 96 weeks, TAF caused less BMD decline, with reductions of 1.57% to 1.90% at the LS and 2.00% to 2.66% at the TH, whereas TDF caused significantly more BMD loss. These findings suggest that switching from TDF to TAF may improve bone health outcomes in PLHIV. The study also found that protease inhibitors (PIs) and ART-naïve patients initiating TDF treatment experienced greater BMD losses, indicating that early ART decisions significantly impact long-term bone health [155]. While the adverse effects of specific drugs such as TDF on bone health are well established, there is significant variability in the degree of bone loss associated with different drug classes within cART regimens. To provide a clearer understanding of the relationship between these drug classes and their specific mechanisms of bone loss, Table 2 outlines key findings from recent studies (Figure 2).

#### 3.2.2. Clinical and Diagnostic Assessment

Post-menopausal WLHIV face unique clinical challenges that require specialised medical attention. When they present to hospitals, these women exhibit a combination of complications arising from both their HIV status and menopausal changes [116]. They are more likely to present with co-morbidities that lead to loss of bone minerals, such as metabolic dysfunction, including diabetes mellitus, insulin resistance, and CVD [158]. Such individuals are also more likely to present with HCV co-infection. Additionally, vitamin D deficiency, prevalent among PLWHIV, significantly contributes to bone health problems, as discussed above [159,160].

Social factors also play a crucial role in bone health management. Women living in different strata of socioeconomic accessibility may experience a variety of barriers when accessing treatment, including transport to the clinic/hospital, the cost of treatment, the inability to attend appointments due to inflexible shift patterns at work, a loss of income due to disability, social stigma, and a lack of awareness of the importance of clinical treatment and follow-up. 

PLWHIV have historically struggled with modifiable risk factors such as smoking, alcohol, and drug addiction, thereby increasing the risk of bone loss [161]. Smoking and chronic alcohol use are especially detrimental, reducing BMD and increasing the risk of fractures by approximately 60% compared to non-smokers [162,163]. Addressing these issues requires clinicians to take a holistic approach, including an understanding of addiction, awareness of local support groups who specialise in work with PLWHIV, and a culturally sensitive manner of addressing these unique issues. Table 3 summarises the key modifiable risk factors that clinicians should pay attention to and aim to address during patient contact. 

Effective screening and diagnostic tools are essential for monitoring and managing bone density in WLHIV. DXA scans are the gold standard for measuring BMD [168]. The FRAX tool evaluates fracture risk but is likely to underestimate it in HIV-positive patients, as it does not account for HIV-specific factors, so utility of DEXA scans may be more accurate in these instances [109]. 

Studies have found that the FRAX score often underestimates fracture risk in this population [169,170,171,172]. This is largely due to the tool not accounting for HIV as a secondary cause of osteoporosis. PLWH often have an increased prevalence of bone mineral density (BMD) loss due to long-term antiretroviral therapy (ART) and an over-representation of traditional risk factors, such as smoking, vitamin D deficiency, and low body mass index (BMI) [170,171,172]. For instance, Mazzitelli et al. (2021) found that only a small fraction of PLWH with DXA-confirmed osteoporosis had FRAX scores above the 10% threshold, showing poor sensitivity in identifying high-risk individuals [172]. One of the key shortcomings of FRAX in this context is its lack of consideration for the unique biological and clinical factors affecting PLWH. ART, particularly treatments involving tenofovir and protease inhibitors, accelerates bone loss, and HIV itself may exacerbate bone demineralisation through chronic immune activation and inflammation [172]. McGee and Cotter (2023) noted that even when HIV-related factors are included, the FRAX tool does not adequately reflect the true fracture risk unless BMD data are used. However, in resource-limited settings where routine BMD testing may not be feasible, relying on FRAX alone could lead to missed diagnoses and inadequate preventive measures for those at higher risk [171].

To address these limitations, researchers have suggested modifications to the FRAX tool or the use of alternative or complementary risk assessment methods. Mazzitelli et al. (2021) proposed incorporating HIV as an automatic risk factor for secondary osteoporosis in FRAX calculations, as well as routinely using BMD data in the assessment [172]. In addition to FRAX, McGee and Cotter (2023) highlighted the importance of integrating tools that assess frailty and fall risk, given that PLWH, particularly those ageing with the condition, often face a higher risk of falls [171]. Furthermore, Vizcarra et al. (2024) emphasised that using complementary measures, such as the trabecular bone score (TBS), could provide more comprehensive insights into bone health by evaluating bone microarchitecture, which is not captured by FRAX [170].

Ultimately, early screening and intervention are essential to reduce fracture risk in PLWH. Given the limitations of FRAX, a more holistic approach combining multiple tools may be necessary for more accurate risk stratification. For younger individuals and those on long-term ART, routine BMD assessments alongside modified FRAX scores may better identify those needing intervention. Implementing strategies to optimise bone health early, particularly in PLWH, could mitigate the elevated risk of fractures and improve overall quality of life as they age.

WLHIV have lower BMD at the spine, hip, and radius compared to women without HIV, with the largest difference at the spine [148]. The difference in BMD between pre-/early perimenopausal and late peri/post-menopausal women with HIV is larger than in women without HIV, particularly at the radius and spine [173]. 

The prevalence of vertebral fractures is particularly elevated, with studies indicating an odds ratio of approximately 1.7, translating to a 70% higher risk compared to HIV-negative women [174,175]. Additionally, femoral neck fractures occur in about 18% of HIV-positive post-menopausal women, while spinal fractures are present in 32% of this population [176]. Beyond vertebral and femoral neck fractures, other fragility fractures such as those of the wrist and hip are also more common in HIV-positive post-menopausal women. These women experience an overall fracture incidence rate that is 1.5 to 1.9 times higher than the general population [177]. A study in Spain examined fracture risk in people living with HIV. Out of over 1.1 million participants, 0.22% had HIV/AIDS. HIV increased the risk of hip fractures by almost five times. The fracture risk was higher in those aged 59 and over [178].

### 3.3. Challenges and Tribulations Facing Post-Menopausal WLHIV in Low-Income Countries (LICs)

Table 4 contains important findings from studies in LICs; the main studies, such as systematic reviews, clinical trials, and main population studies, have been included. 

As demonstrated in Table 4, Black WLHIV in South Africa experience lower BMD at critical sites, such as the distal radius, with 2.8% of virologically suppressed Black women developing osteoporosis. Additionally, menopause is significantly linked to lower BMD, particularly in the femoral neck [23]. In Thailand, age and lower BMI are strongly associated with reduced BMD in the lumbar spine, total hip, and femoral neck [179]. In India, menopause is a major factor contributing to low BMD, with earlier menopause and lower T-scores in the lumbar spine and femoral neck being prevalent among Indian WLHIV [180].

Brazilian WLHIV on long-term antiretroviral therapy (ART) show a significantly earlier onset of menopause and higher FRAX scores, indicating an elevated fracture risk [40]. A longitudinal study in Soweto, South Africa, found significant bone loss in post-menopausal WLHIV, with increased osteoporosis risk after adjustments for HIV status [181].

Across sub-Saharan Africa, ethnic-specific FRAX models reveal a high risk of osteoporosis in older WLHIV. Key risk factors include HIV, malnutrition, and chronic inflammation (“inflammaging”). Limited access to diagnostic tools such as DEXA scans and low awareness of bone health issues further exacerbate this problem in resource-limited settings [182,183,184,185,186,187,188,189,190,191].

Post-menopausal bone loss is a significant health concern in LICs. For example, the prevalence of osteoporosis in Africa ranges from 18.2% to 65.8% [189] across sub-Saharan Africa. Studies from Kenya show high rates of osteoporosis among peri-urban women, worsened by limited access to diagnostics and healthcare [190]. Similarly, research from Morocco, Tunisia and sub-Saharan Africa reflects a high prevalence of osteopenia and osteoporosis, with poor nutrition and healthcare access exacerbating the issue [191].

Moreover, the majority of bone mineral density in Africa was measured using ultrasound due to the limited availability of DEXA scans, which may have underestimated the true prevalence of osteoporosis [189]. A recent call to action for osteoporosis research in sub-Saharan Africa has demonstrated that the prevalence of osteoporosis and fragility fracture incidence is higher than previously researched and that there are large gaps in research that consider Africa as a whole [182,183,184,185,186,187,188,189]. 

Most of the HIV research has concentrated on urban areas, particularly in South Africa [23]. This urban-centric focus leaves a substantial gap in understanding the prevalence of HIV and osteoporosis, the associated risk factors, and its impacts in rural regions within Africa and other LICs. Rural populations often have different lifestyle factors, healthcare access issues, and socio-cultural dynamics compared to their urban counterparts, which can influence the onset and progression of post-menopausal osteoporosis [192]. A recent systematic review demonstrated that, in LICs, individuals living in urban areas were likely to have higher BMD [193]. For example, using a Chinese study population of 490 males and 689 females of ages 50–70, Gu et al. showed that urban men and women had significantly higher BMD than their rural counterparts (*p* < 0.01). This may be likely due to improved access to healthy food, education, and healthcare in urban settings [194]. In contrast, in higher-income countries (HICs), individuals living in rural areas have been found to be more likely to have higher BMD. For example, a Norwegian study consisting of 10,667 male and female participants with ages ranging between 40 and 75 exhibited higher BMD in rural populations compared to urban ones. This may be due to increased physical activity, more individuals within healthy BMI ranges, and access to less processed foods that may contain greater nutritional benefits [195]. Therefore, whether fracture risk is higher in rural or urban areas may vary according to a country’s economic development. LICs may follow the trend of HICs as they experience continued economic growth.

The lack of comprehensive data from these rural groups hampers the development of tailored interventions that could address their specific needs and improve bone health outcomes for post-menopausal women living in these areas.

WLHIV in rural regions often have low awareness of the importance of treatment and regular health follow-ups for bone health [182]. This lack of knowledge can lead to the delayed diagnosis and treatment of osteoporosis, increasing the risk of fractures and other complications. The healthcare infrastructure in rural areas is typically less developed, with fewer diagnostic facilities available. There are limited specialists available with an interest in bone health. For example, in 2022, there were 30 rheumatologists serving 200 million patients in Nigeria and 2 rheumatologists serving 28 million patients in Ghana [196]. 

This situation is compounded by logistical challenges such as long distances to healthcare facilities, inadequate transportation options, and difficult road conditions, making it difficult for women to access necessary medical care [197]. Consequently, many women may not receive timely interventions that could prevent or mitigate bone loss. Moreover, different WLHIV may have access to different health resources in neighbourhoods, leading to variances in awareness of the importance of medical follow-up and access to further healthcare [198].

In addition to logistical barriers, WLHIV in LICs face significant social and cultural challenges in managing their health. Social stigma and cultural shame associated with seeking medical help for conditions perceived as sex-related, age-related, or non-urgent can discourage women from pursuing treatment [199]. The cultural stigma surrounding HIV and conditions perceived as sex-related or age-related discourages many women from seeking necessary medical care [200,201].

Compounding these barriers is the prevalent use of traditional medicine, which often coexists with modern healthcare practices. Some communities rely on traditional healers, which can delay or prevent the uptake of modern medical treatments, including antiretroART [202]. Furthermore, responsibilities such as childrearing and household management, often combined with agricultural or informal work, limit women’s time and resources spent on their health [203]. Communities in LICs often depend on locally sourced foods, which can provide nutritional benefits that help manage HIV. However, the availability and variety of these foods are often insufficient to meet the nutritional needs of WLHIV [202].

The economic impact of losing work time to seek medical care can be substantial, further deterring women from accessing healthcare services. A recent cross-sectional study set in South Africa consisting of 996 participants found that individuals in rural areas were more likely to ask relatives and neighbours for assistance with money and food compared to their urban counterparts [204]. These multifaceted barriers highlight the need for targeted awareness campaigns, community-based interventions, and healthcare policies that address the unique challenges faced by post-menopausal women in LICs.

Research suggests that built environments (spaces in which people live and work) influence health behaviours [205]. The resources and road conditions in the neighbourhood can give residents the opportunity to seek medical attention, and this leads to more accessibility of HIV testing facilities and more screening and early detection of HIV and the prevention of complications [206]. It is crucial to remember that stigma and discrimination related to HIV may discourage locals from utilising adjacent HIV health services [207]. Studies have shown that, in LICs, poor road conditions and limited transportation options create significant barriers for people, particularly WLHIV, to reach healthcare facilities. Proximity to healthcare centres is directly associated with higher rates of HIV testing and treatment adherence, while more distant or harder-to-reach clinics are linked to lower service utilisation and delayed diagnosis [208]. Additionally, community characteristics, such as the availability of healthcare resources and social support networks within neighbourhoods, have been found to significantly influence the accessibility of HIV services. Areas with better healthcare infrastructure, including more accessible HIV testing facilities, foster earlier detection of HIV, improved ART adherence, and better overall health outcomes [209]. To improve access, targeted interventions such as mobile health services and improving road infrastructure can significantly enhance the ability of people to seek timely medical attention. Public health policies that focus on improving the built environment and increasing the accessibility of health services can make a significant difference in HIV care and prevention in LICs [208,209].

In HICs, diagnostic tools such as DEXA scans are widely accessible, allowing for routine screening of osteoporosis, particularly among at-risk groups such as WLHIV. Early detection is crucial in preventing severe bone loss and reducing fracture risks [210,211]. Conversely, LICs suffer from limited access to advanced diagnostic technologies. Many rural areas in LICs rely on heel quantitative ultrasound, which, although cost-effective, is less accurate and may underestimate the prevalence and severity of bone loss [212,213]. This diagnostic gap highlights the need for portable and affordable diagnostic tools, such as mobile DEXA units, to enhance early detection and intervention in LICs [211].

Heterosexual women have been found to experience greater disclosure shame compared to heterosexual males and non-heterosexual females [214]. Therefore, disclosure of shame may affect healthcare-seeking behaviour in post-menopausal WLHIV. Additionally, a cross-sectional study comparing 64 LICs and middle-income countries found that HIV stigma was lower in countries with greater gross domestic product per capita [215]. HIV stigma was also lower in men and those who had adequate knowledge.

**Treatment Options and Availability:** HICs benefit from ART, such as tenofovir alafenamide (TAF), which is bone-sparing compared to older regimens such as tenofovir disoproxil fumarate (TDF). This has significantly reduced osteoporosis-related complications in WLHIV in HICs [210,213]. However, in LICs, the continued reliance on TDF due to its lower cost exacerbates bone loss, particularly in older women who are already vulnerable due to HIV-related factors. Additionally, access to osteoporosis treatments such as bisphosphonates, calcium, and vitamin D is more widespread in HICs, further improving bone health outcomes [211,212].

**Healthcare Infrastructure:** The healthcare systems in HICs are better equipped to manage osteoporosis and other bone health issues in HIV-positive populations. Public health campaigns, regular screenings, and easy access to specialised care ensure that WLHIV receive timely and effective treatment [216]. In contrast, LICs face significant shortages in terms of healthcare providers, particularly specialists such as rheumatologists, resulting in delayed or suboptimal care [213]. Rural populations are particularly disadvantaged due to long distances to healthcare facilities, limited transportation options, and a lack of necessary medical expertise [211,213].

**Potential Interventions:** Addressing these disparities requires several interventions. First, expanding the availability of bone-sparing ARTs such as TAF in LICs would help reduce bone loss among WLHIV. Additionally, community-based educational programs to raise awareness about osteoporosis, along with mobile healthcare units or telemedicine solutions, could provide critical care to women in rural and underserved regions. Ensuring the availability of diagnostic tools and treatments, such as calcium and vitamin D supplements, would also improve outcomes [210,211,216].

#### 3.3.1. Management

As discussed above, the unique population of post-menopausal WLHIV requires a tailored approach consisting of non-pharmacological and pharmacological management.

**Non-pharmacological management:** The following aspects of care play a key role in the non-pharmacological management of bone fracture risk in post-menopausal WLHIV:

- Education and awareness of premature menopause and associated risks of bone loss. Education can help prevent premature menopause by promoting healthier lifestyle choices, such as reducing smoking and improving nutrition, which are key risk factors for early menopause [217]. Educating women about the importance of vitamin D and calcium intake can delay menopause and improve health outcomes. Additionally, increased awareness of early medical intervention options, such as hormone replacement therapy, enables earlier care, reducing the risk of complications from premature menopause [218].
- Encouragement of regular exercise, particularly resistance and aerobic training [102].
- Tailored help to address smoking, alcohol, and drug use.
- Measurement of calcium and vitamin D levels for those at high risk of fracture due to co-morbidities, previous fracture, or family history.
- Use of DXA scans for all post-menopausal women with regular follow-up [219].
- Population-specific FRAX tools, in particular for women living in low-income countries [220].
- Clinical recognition of nutritional requirements of patients in this population–malnourished versus those with diabetes.
- Social and mental health impact of disease: specialised culturally-sensitive talking services addressed to WLHIV.
- Joint HIV, bone health, and menopause clinics and MDTs to develop tailored plans.

**Pharmacological management:** The following aspects of care play a key role in the pharmacological management of bone fracture risk in post-menopausal WLHIV:

- Personalised cART regimes that balance bone loss at an individual level [221].
- The addition of bisphosphonates when stopping TDF-based therapy has been shown to increase BMD [222].
- HRT use has been shown to increase BMD [223].
- Romosozumab, teriparatide, and denosumab have shown benefit in post-menopausal women [110,153].
- Abaloparatide (a synthetic peptide analogue of parathyroid hormone-related protein (PTHrP)) is used to manage and treat osteoporosis. Recently, the National Institute for Health and Care Excellence (NICE) recommended abaloparatide as an option for treating osteoporosis in women with menopause, only if they have a very high risk of fracture (https://www.nice.org.uk/guidance/ta991 (accessed on 11 August 2024)).

#### 3.3.2. Future Directions

Further research is needed to elucidate the intricate interactions between menopause, HIV, and bone health specifically. Currently, most long-term HIV-related studies have explored the impact of the virus on combined populations of males and females, or exclusively males. Most of the HIV literature focused on the female population has explored the transmission and effects of HIV during pregnancy and the postpartum period [101,102]. Therefore, there are stark gaps in the research focused on WLHIV. Long-term longitudinal studies investigating the long-term effects of cART on bone density in post-menopausal women, the impact of lifestyle modification metabolic changes such as fatty liver, obesity, and diabetes on the bone health of post-menopausal WLHIV, and the efficacy of various interventions in this population are crucial, especially with newer potential therapies becoming available, such as abaloparatide [153,154,155]. Additionally, exploring the genetic and molecular mechanisms underlying the susceptibility to bone loss in HIV-positive post-menopausal women could pave the way for personalised therapeutic approaches, as menopause is a pivotal life event and is experienced in a unique manner by individuals. Furthermore, studies investigating the social and mental health impact of early menopause and fractures in WLHIV need to take place [156]. As we suggested before, metabolic clinics for PLWHIV represent an essential part in the treatment of cardiovascular disease and metabolic bone disease [224].

Future research should be more targeted and specific. To address current knowledge gaps, long-term cohort studies are essential. These should focus on the impact of different antiretroviral therapies, such as comparing bone-sparing therapies such as tenofovir alafenamide (TAF) with older regimens, such as tenofovir disoproxil fumarate (TDF), in WLHIV. This would help identify optimal treatments to minimise bone loss.

Additionally, intervention trials should examine the effects of lifestyle changes, such as diet, physical activity, and management of metabolic conditions such as diabetes or obesity, on bone density in this population. Such studies should evaluate both pharmacological and non-pharmacological strategies, potentially testing newer drugs such as abaloparatide alongside lifestyle modifications.

Genetic and molecular research is another priority area, with studies needed to explore how HIV accelerates bone loss during menopause. These findings could guide the development of personalised therapies. Lastly, qualitative research into the mental and social impacts of early menopause and fractures in WLHIV is important to inform holistic care approaches, including the integration of bone health services in HIV care clinics.

Research should further explore the role of nutrition and access to nutrient-dense foods in this population, particularly given that HIV-related gastrointestinal complications and nutrient malabsorption can worsen deficiencies in essential nutrients, such as calcium, vitamin D, and others vital for bone health. Incorporating access to locally sourced, nutritious foods and educating WLHIV on nutritional strategies could be an integral part of their HIV care.

#### 3.3.3. Limitations and Strengths

The limitations of our narrative review include the broad search across different research databases. This is not a systematic review or meta-analysis, as the focus is on the entire topic, which may lead to some variation in the types of studies included. Another limitation is that we have only included studies published in the English language. The strengths of our review include the use of a variety of studies from different geographical locations, allowing for a richer and more nuanced perspective on the topic. This review sets the stage for current research evidence available on post-menopausal women living with HIV (WLHIV) who are at risk of bone fractures. It has allowed specific gaps in the literature to be identified and has provided direction for future research. Importantly, the review covers data from both developed and developing countries, enabling health professionals in developing countries to establish health policies that will help narrow the gap in terms of health services. A unique strength of this review is that it provides robust evidence for why “an initiative to promote the concept of effective menopausal care among women living with HIV” is needed in order to improve bone health. Additionally, the narrative style of the review may make it useful for a wider audience, including clinicians, policymakers, postgraduate and undergraduate students, and laypeople.

## 4. Conclusions

Menopause and HIV infection independently contribute to significant bone loss and increased fracture risk. The convergence of these conditions in post-menopausal women living with HIV exacerbates bone health issues, necessitating a multifaceted approach to management. Optimising cART regimens, implementing lifestyle modifications, and providing individuals with tailored clinical care are key strategies to mitigate the adverse effects on bone health. Ongoing research and tailored interventions will be essential in improving outcomes for this vulnerable population.

## Figures and Tables

**Table 1:** Locations of different types of fractures in post-menopausal women with HIV.

Study Type	Fracture Site	Findings
Cross-sectional [42]	Lumbar Spine, Femoral Neck, Total Hip	WLHIV had a 6.3% greater incidence of at least one vertebral fracture. WLHIV had lower femoral neck BMD (*p* = 0.012) and lower total hip BMD (*p* = 0.041). 22/104 WLHIV had osteoporosis. However, they were not found to be on any treatment.
Population-based cohort [43]	Hip	41.9% of the population aged 65 and above was female. HIV status was not associated with increased hip fracture risk: HR 1.02, 95% CI 0.78–1.34 *p* > 0.05.
Systematic review and meta-analysis [44]	Vertebral	PLHIV had an odds ratio of vertebral fractures of 2.3 (95% CI 1.37, 3.85, I^2^ 98.2% *p* < 0.00001) when compared to non-infected individuals.
Population-based cohort [45]	Hip	Older patients with HIV were more likely to experience a hip fracture: HR 2.11 [1.05–4.22], *p* = 0.035).
Population-based cohort [12]	Hip, Vertebral	FRAX-estimated risk for hip and major osteoporotic fractures was statistically higher in PLWH (*p* < 0.001).
Cross-sectional [23]	Radius	Post-menopausal WLHIV had a greater risk of lower BMD and risk of fracture at the proximal and distal radius (*p* = 0.048)

**Table 2:** Comparison of Antiretroviral Drug Classes and Their Impact on Bone Mineral Density (BMD) in Patients with HIV.

ART Class	Key Drugs	Mechanism of Bone Loss	BMD Impact Findings	Source(s)
Nucleoside Reverse Transcriptase Inhibitors (NRTIs)	TDF (Tenofovir Disoproxil Fumarate), TAF (Tenofovir Alafenamide)	- TDF linked to renal phosphate wasting, causing decreased calcium absorption and direct suppression of bone formation.	- TDF leads to significant BMD loss, particularly in the spine and hip. - Switching from TDF to TAF results in BMD recovery due to reduced renal and bone toxicity.	[156]
Non-Nucleoside Reverse Transcriptase Inhibitors (NNRTIs)	Efavirenz (EFV), Nevirapine	- Efavirenz reduces vitamin D levels by inducing liver enzymes, impairing calcium absorption, and leading to bone demineralization.	- Efavirenz use is associated with bone loss, especially when combined with TDF. - Vitamin D supplementation can mitigate some of the bone loss associated with EFV.	[156,157]
Protease Inhibitors (PIs)	Atazanavir, Darunavir	- PIs may indirectly activate osteoclasts through increased inflammation and disrupt calcium metabolism.	- PIs cause moderate BMD reductions, but less severe than TDF. - When combined with TAF, PIs show less impact on bone health than when combined with TDF.	[156,157]
Integrase Strand Transfer Inhibitors (INSTIs)	Dolutegravir (DTG), Raltegravir (RAL), Elvitegravir (EVG)	- INSTIs generally have a neutral or positive effect on BMD, with no significant negative impact on bone health.	- Meta-analyses demonstrate better BMD preservation with INSTI-based regimens, especially when combined with TAF.	[156,157]
Combination Therapy	TDF/EFV, TAF/DTG	- TDF/EFV combinations lead to greater bone loss due to both TDF-induced phosphate wasting and EFV-induced vitamin D deficiency.	- Switching from TDF/EFV to TAF/DTG results in significant improvements in BMD, making it a preferred switch for patients with osteoporosis or at risk.	[156,157]

**Table 3:** A Summary of Modifiable Risk Factors Leading to Bone Loss in Post-Menopausal WLHIV.

Risk Factor	Description
Knowledge, attitude, and practice	Ignorance and misconception about menopause. There is a need to increase knowledge and attitude and practice of WLHIV with regard to how to deal with menopause symptoms and protect themselves from risk of fractures [164].
Compliance with medication	Ensure patients have adequate compliance with medication. Aim to discuss compliance at every health check-up or point of contact [164].
Nutrition	Poor nutritional intake has been demonstrated in WLHIV, leading to vitamin D, calcium, magnesium, and phosphorus deficiencies [164]. Nutritional deficiencies in WLHIV are caused by malabsorption due to HIV and ART and worsened by food insecurity in low-income areas, leading to poor access to nutrient-rich diets [165,166].
Smoking	Smoking increases the risk of osteoporosis and fractures and is more common among HIV-positive individuals [164,167].
Alcohol use	Excessive alcohol consumption negatively impacts bone health and is a risk factor for low BMD [164,167].
Physical activity	Exercise, including combined resistance and aerobic training, can slow down bone loss in individuals with HIV [164].
Obesity	Obesity is a modifiable risk factor that can increase bone loss in individuals with HIV [164].

**Table 4:** Summary of osteoporosis and bone loss in WLHIV in sub-Saharan Africa, Thailand, Western India, and Brazil.

Study Source	Study Type	Population	Findings
[23,40,179,180]	Cross-sectional	Perimenopausal women in South Africa, HIV-infected, cART-naïve individuals in South Africa, Women in NorthWest province, South Africa PLHIV who are older than 50 years of age in Thailand PLHIV in western India who were on long-term cART vs. cART-naïve Brazilian post-menopausal WLHIV on cART living in the Amazon	In black South African women, menopause is associated with lower BMD at the distal radius and lower cortical density at the proximal radius; 2.8% of virologically suppressed Black females had osteoporosis [23]. In Thai WLHIV, age and lower BMI were significantly associated with lower BMD at the lumbar spine, total hip, and femoral neck [179]. In Indian WLHIV, menopause is strongly associated with low BMD (*p* = 0.002) [180]. In Brazilian WLHIV, age of menopause onset is significantly earlier (*p* < 0.001). FRAX score was higher in WLHIV (*p* < 0.001). Lower T scores in lumbar spine and femoral neck following menopause onset was also seen in Brazilian WLHIV [40]. Older HIV-positive women with low educational status are at higher risk [156].
[181]	Longitudinal (5 years)	450 women aged 40–60 in Soweto, South Africa	Significant bone loss during menopause in women with and without HIV [181]. HIV-infected women had greater bone loss after adjustments [181].
[182,183,184,185,186,187,188,189]	Review	Post-menopausal women in South Africa, rural South Africa, sub-Saharan Africa, resource-limited settings in South Africa, African nations using cART, various African regions and ethnic groups (mainly in the Gambia, Nigeria, Kenya, and Cameroon)	Ethnicity-specific FRAX models for South Africa available for use in HIV clinics. DEXA is the gold standard for BMD but is limited despite need for routine assessment in HIV clinics. Limited access to diagnostic and treatment options [182,183]. Osteoporosis more common than sarcopenia. Higher bone loss in older women with HIV. Key risk factors: HIV, malnutrition, ‘inflammaging’ [184]. Limited access to diagnostic and treatment options [185]. Fracture rates expected to rise due to HIV and cART [186,187]. Younger individuals (<60 years) more affected [186]. Heel quantitative ultrasound is a cost-effective alternative [187,188,189]. Tenofovir Alafenamide Fumarate (TAF) is more bone-sparing than TDF but expensive [187,188,189]. Need for awareness of BMD decreases with TDF [187,188,189]. Osteoporosis and fracture rates vary across Africa [187,188,189]. Low awareness among population and health authorities [187,188,189].

## Data Availability

This review article and all information available included in this review.
