# Correction: Jamshaid et al. Bone Loss and Fractures in Post-Menopausal Women Living with HIV: A Narrative Review. *Pathogens* 2024, *13*, 811

**DOI:** 10.3390/pathogens14111118

**Published:** 2025-11-03

**Authors:** Maryam Jamshaid, Amirmohammad Heidari, Ahmed Hassan, Dushyant Mital, Oliver Pearce, Maria Panourgia, Mohamed H. Ahmed

**Affiliations:** 1 Department of Trauma and Orthopaedics, Liverpool University Hospital NHS Trust, Liverpool L69 3BX, UK; maryamjamshaid9819@gmail.com (M.J.);; 2 School of Medicine, University of Liverpool, Liverpool L69 3BX, UK; 3 Faculty of Medicine, Alexandria University, Alexandria 21500, Egypt; ahmed.mohamed2133@alexmed.edu.eg; 4 Department of HIV and Blood Borne Virus, Milton Keynes University Hospital NHS Foundation Trust, Eaglestone, Milton Keynes MK6 5LD, UK; dushyant.mital@mkuh.nhs.uk; 5 Department of Trauma and Orthopaedics, Milton Keynes University Hospital NHS Foundation Trust, Milton Keynes MK6 5LD, UK; oliver.pearce@mkuh.nhs.uk; 6 Department of Geriatric Medicine, Milton Keynes University Hospital NHS Foundation Trust, Eaglestone, Milton Keynes MK6 5LD, UK; maria.panourgia@mkuh.nhs.uk; 7 Faculty of Medicine and Health Sciences, University of Buckingham, Buckingham MK18 1EG, UK; 8 Department of Medicine and HIV Metabolic Clinic, Milton Keynes University Hospital NHS Foundation Trust, Eaglestone, Milton Keynes MK6 5LD, UK

In the original publication [1], errors were identified in Figure 1, Table 4, and several reference citations throughout the manuscript. These errors occurred because, during the exchange of manuscripts between the authors, some of the intended corrections were unfortunately not implemented. This oversight was noticed during the publication process. To ensure the accuracy of the published information, the authors deemed it necessary to issue the following corrections.

## Changes to Figure 1

The following corrections have been made to the “Breakdown of studies” section in Figure 1:

“Review articles 78” has been corrected to “Review articles 79”.

and “Included” section in Figure 1:

“Studies included in the review (n = 223)” has been corrected to “Studies included in the review (n = 224)”;

The corrected Figure 1 appears below.

## Changes to Table 4

Multiple reference citations in Table 4 have been corrected in both the “Findings” and “Study Sources” columns. The corrected reference numbers now accurately reflect the appropriate citations as listed in the bibliography. The corrected Table 4 appears below.

## Text Corrections

Several reference citations throughout the manuscript have been updated to ensure accuracy and proper correspondence with the reference list. Notably,

On page 23, the reference citation [153] in the paragraph discussing abaloparatide has been replaced with the direct URL citation (https://www.nice.org.uk/guidance/ta991, (accessed on 11 August 2024).) to provide readers with direct access to the NICE guidance.

On page 24, the reference citation “[157–164]” has been corrected to “[224]” to accurately reflect the appropriate source.

## Reference Updates

Two references in the bibliography have been updated:

Reference 184 has been replaced with: SeyedAlinaghi, S.; Ghayomzadeh, M.; Mirzapour, P.; Maroufi, S.F.; Pashaei, Z.; Ali, Z.; Tantuoyir, M.M.; Aghaie, N.; Vahedi, F.; Salmani, R.; et al. A Systematic Review of Sarcopenia Prevalence and Associated Factors in People Living with Human Immunodeficiency Virus. *J. Cachexia Sarcopenia Muscle* **2023**, *14*, 1168–1182. https://doi.org/10.1002/jcsm.13212.

Reference 188 has been replaced with: Adejumo, O.A.; Malee, K.M.; Ryscavage, P.; Hunter, S.J.; Taiwo, B.O. Contemporary Issues on the Epidemiology and Antiretroviral Adherence of HIV-Infected Adolescents in Sub-Saharan Africa: A Narrative Review. *J. Int. AIDS Soc.* **2015**, *18*, 20049. https://doi.org/10.7448/ias.18.1.20049.

A new reference (224) has been added: Ahmed, M.H.; Woodward, C.; Mital, D. Metabolic Clinic for Individuals with HIV/AIDS. *Cardiovasc. Endocrinol.* **2017**, *6*, 109–112. https://doi.org/10.1097/xce.0000000000000128.

With this correction, the order of some references has been adjusted accordingly.

The authors state that these corrections do not affect the scientific conclusions of the manuscript. This correction was approved by the Academic Editor. The original publication has also been updated.

## Figures and Tables

**Figure 1:**
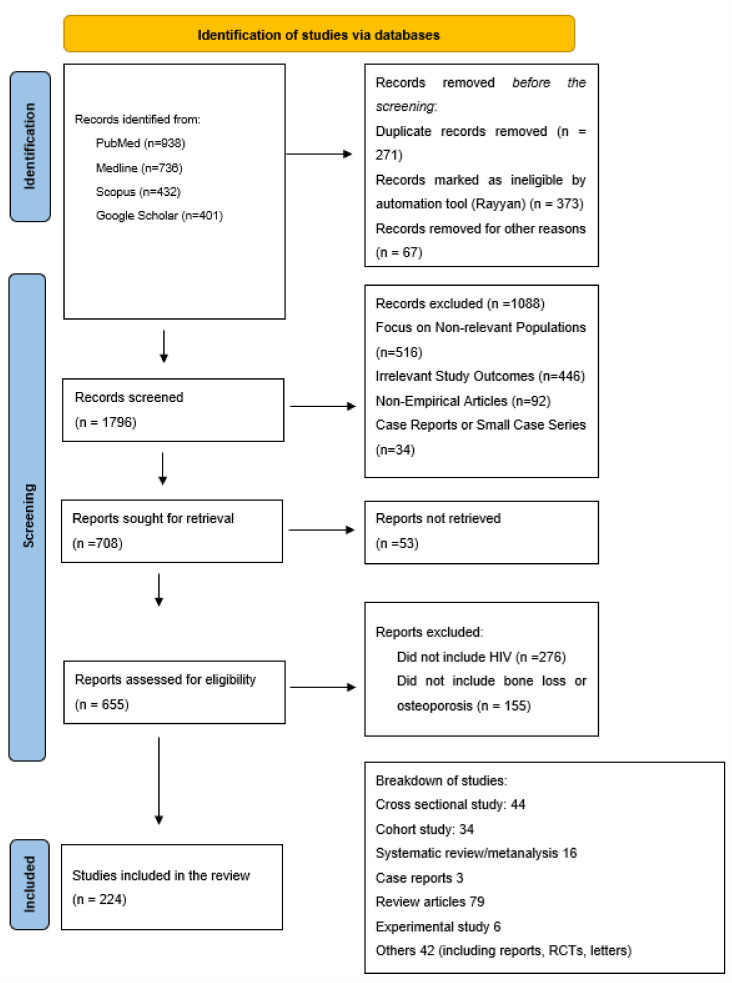
Diagram detailing the identification of studies via databases included in this narrative review.

**Table 4:** Summary of osteoporosis and bone loss in WLHIV in sub-Saharan Africa, Thailand, Western India, and Brazil.

Study Source	Study Type	Population	Findings
[23,40,179,180]	Cross-sectional	Perimenopausal women in South Africa, HIV-infected, cART-naïve individuals in South Africa, Women in NorthWest province, South Africa PLHIV who are older than 50 years of age in Thailand PLHIV in western India who were on long-term cART vs. cART-naïve Brazilian post-menopausal WLHIV on cART living in the Amazon	In black South African women, menopause is associated with lower BMD at the distal radius and lower cortical density at the proximal radius; 2.8% of virologically suppressed Black females had osteoporosis [23]. In Thai WLHIV, age and lower BMI were significantly associated with lower BMD at the lumbar spine, total hip, and femoral neck [179]. In Indian WLHIV, menopause is strongly associated with low BMD (*p* = 0.002) [180]. In Brazilian WLHIV, age of menopause onset is significantly earlier (*p* < 0.001). FRAX score was higher in WLHIV (*p* < 0.001). Lower T scores in lumbar spine and femoral neck following menopause onset was also seen in Brazilian WLHIV [40]. Older HIV-positive women with low educational status are at higher risk [156].
[181]	Longitudinal (5 years)	450 women aged 40–60 in Soweto, South Africa	Significant bone loss during menopause in women with and without HIV [181]. HIV-infected women had greater bone loss after adjustments [181].
[182–189]	Review	Post-menopausal women in South Africa, rural South Africa, sub-Saharan Africa, resource-limited settings in South Africa, African nations using cART, various African regions and ethnic groups (mainly in the Gambia, Nigeria, Kenya, and Cameroon)	Ethnicity-specific FRAX models for South Africa available for use in HIV clinics. DEXA is the gold standard for BMD but is limited despite need for routine assessment in HIV clinics. Limited access to diagnostic and treatment options [182,183]. Osteoporosis more common than sarcopenia. Higher bone loss in older women with HIV. Key risk factors: HIV, malnutrition, ‘inflammaging’ [184]. Limited access to diagnostic and treatment options [185]. Fracture rates expected to rise due to HIV and cART [186,187]. Younger individuals (<60 years) more affected [186]. Heel quantitative ultrasound is a cost-effective alternative [187–189]. Tenofovir Alafenamide Fumarate (TAF) is more bone-sparing than TDF but expensive [187–189]. Need for awareness of BMD decreases with TDF [187–189]. Osteoporosis and fracture rates vary across Africa [187–189]. Low awareness among population and health authorities [187–189].
